# The Proapoptotic Gene *Bad* Regulates Brain Development via p53-Mediated Stress Signals in Zebrafish

**DOI:** 10.3390/cells10112820

**Published:** 2021-10-20

**Authors:** Jo-Chi Hung, Jen-Leih Wu, Huei-Ching Li, Hsuan-Wen Chiu, Jiann-Ruey Hong

**Affiliations:** 1 Laboratory of Molecular Virology and Biotechnology, Institute of Biotechnology, National Cheng Kung University, Tainan 701, Taiwan; betty01269@gmail.com (J.-C.H.); kjs-211722@yahoo.co.jp (H.-C.L.); d5432345@ms19.hinet.net (H.-W.C.); 2 Laboratory of Marine Molecular Biology and Biotechnology, Institute of Cellular and Organismic Biology, Academia Sinica, Nankang, Taipei 115, Taiwan; jlwu@gate.sinica.edu.tw; 3 Department of Biotechnology and Bioindustry Sciences, National Cheng Kung University, Tainan 701, Taiwan

**Keywords:** PCD, *Bad*, brain defect, environmental stress, p53/caspase-8 death signaling, knockdown, genome-wide approach

## Abstract

Studies have shown that the BH3-only domain Bad regulates brain development via the control of programmed cell death (PCD), but very few studies have addressed its effect on the molecular signaling of brain development in the system. In this work, we examined the novel role of zebrafish *Bad* in initial programmed cell death for brain morphogenesis through the priming of p53-mediated stress signaling. In a biological function study on the knockdown of *Bad* by morpholino oligonucleotides, at 24 h post-fertilization (hpf) *Bad* defects induced abnormal hindbrain development, as determined in a tissue section by means of HE staining which traced the damaged hindbrain. Then, genome-wide approaches for monitoring either the upregulation of apoptotic-related genes (11.8%) or the downregulation of brain development-related genes (29%) at the 24 hpf stage were implemented. The p53/caspase-8-mediated apoptotic death pathway was strongly involved, with the pathway being strongly reversed in a *p53* mutant (*p53^M214K^*) line during *Bad* knockdown. Furthermore, we propose the involvement of a p53-mediated stress signal which is correlated with regulating Bad loss-mediated brain defects. We found that some major genes in brain development, such as *crybb1*, *pva1b5*, *irx4a*, *pax7a*, and *fabp7a*, were dramatically restored in the *p53^M214K^* line, and brain development recovered to return movement behavior to normal. Our findings suggest that Bad is required for (PCD) control, exerting a p53 stress signal on caspase-8/tBid-mediated death signaling and brain development-related gene regulation.

## 1. Introduction

In many situations, apoptosis inhibition causes embryonic lethality, developmental abnormalities, and various pathologies [1]. Originally, PCD was almost synonymous with apoptosis. More recently, PCD has been classified into apoptosis (type I cell death), necroptosis (type II), and autophagy-mediated cell death (type III), which are all pathways that require tight regulation [1,2].

Observations of programmed cell death in certain model organisms such as *Caenorhabditis elegans*, *Drosophila melanogaster*, and mice (*Mus musculus*) have shaped our understanding of how cells undergo PCD [1,3,4,5,6,7]. Various models to explain why cells need to die during development have proposed reasons for this effect, including the sculpting and deletion of structures, nutrient supply, the regulation of cell number, and the elimination of abnormal cells [8,9,10].

The BCL-2 family of proteins constitutes a critical apoptotic control point residing immediately upstream of the mitochondria. Antiapoptotic members display sequence conservation throughout all four BCL-2 homology domains (BH1–BH4) [2,11]. BH3-only molecules, including BAD, BID, NOXA, PUMA, BIK, and BIM, operate upstream, connecting proximal death signals to the activation of BAX, which permeabilizes the outer mitochondrial membrane to release cytochrome *c* [12]. BAD was the first BH3-only molecule to be connected to proximal signal transduction through differential phosphorylation in response to extracellular survival factors [13,14]. Dephosphorylated BAD appears to be active and binds to BCL-2 and BCL-XL at the mitochondria; however, when phosphorylated at serine sites (Ser-112, -136, and -155), BAD is inactive and can be bound by 14-3-3 [14,15]. Factors including interleukin-3 (IL-3), insulin-like growth factor 1 (IGF-1), and nerve growth factor transduce intracellular survival signaling by activating kinase cascades that phosphorylate death substrates including BAD. The phosphatidylinositol 3-kinase (PI3K) pathway, including (but likely not restricted to) AKT and p70S6K, mitochondrial-tethered protein kinase A (PKA), and RSK, has been implicated in BAD phosphorylation [16,17,18,19,20,21]. In a loss-of-function approach, proapoptotic BAD suppresses tumorigenesis in the lymphocyte lineage [22]. BAD overexpression in zebrafish induces apoptosis both in vitro and in vivo, which may have biological implications for apoptosis during zebrafish development [23]. Bid is a proapoptotic BH3-only domain protein of the Bcl-2 family [9]. Upon stimulation of death receptors such as TRAIL receptors or CD95, the activation of caspase-8 at the death-inducing signaling complex (DISC) results in the proteolytic processing of Bid into tBid [24]. tBid, in turn, translocates to the mitochondria to promote the activation of Bax and Bak, cytochrome c release into the cytosol, and caspase activation, thus connecting the extrinsic pathway to the intrinsic pathway of apoptosis [24].

To date, two major fish systems have been used in model animal systems. The zebrafish is the canonical fish model for studying human disease. A large and steadily growing community relies heavily on this fish due to its excellent features, which have been highlighted in many reviews [25,26,27,28,29]. Although much less well-known and used only by a small but steadily growing, community of researchers, the medaka *Oryzias latipes* (also known as the Japanese ricefish) can be regarded as a complementary model, and is equivalent in many ways to the most common fish model [30]. On the contrary, other fish models have been mentioned [31], but few are widely used.

Zebrafish possess several advantages over rodent models in the study of vertebrate development and disease. These include hundreds of embryos in a single clutch and optical clarity of the developing embryo, which allows live imaging at the organism level [32,33]. In addition, the use of tissue-specific transgenic animals can be easily performed under the control of various selected gene promoters. Recent improvements in the Tol2-based transgenic system in zebrafish [34] have allowed the control of gene expression in a spatiotemporal manner by coupling with regulatory elements such as GAL4/UAS, Cre/LoxP, and CRISPR [35,36,37]. These advantages allow live imaging of cells and the tracking of cellular dynamics in vivo to study the underlying molecular mechanisms of various developing organs. This possibility was clearly shown in recent studies [38,39,40,41], in which drugs that had been approved for alleviating metabolic syndromes in humans were also effective in a zebrafish model.

Morpholinos (MOs) are chemically modified oligonucleotides with base-stacking abilities similar to those of natural genetic material [42]. Morpholinos have been shown to bind to and block the translation of mRNA during cell genesis in zebrafish [43].

Recently, we found that knockdown of the BH3-only molecule Bad correlates with the upregulation of both apoptotic and oxidative stress genes. Furthermore, this *Bad* knockdown-mediated environmental stress can further influence normal cell migration in the formation of the three germ layers (especially the ectoderm) for further brain development [44], but very few studies have addressed Bad function in the molecular mechanisms of brain embryonic development.

In a zebrafish system, we identified a new role for the BH3-only domain Bad in triggering PCD during early embryonic development, related to the completion of the development of tissues or organs such as the brain and affecting some novel gene expression during early development. These functions and the relationship between p53-mediated stress signaling on the caspase-8/tBid cell death pathway and brain development-related regulation should all be further addressed.

## 2. Materials and Methods

### 2.1. Experimental Fish

The wild-type AB strain was used for MO injection. The AB strain of zebrafish (*Danio rerio*), the *tp*53^M214K^ mutant [45] line, and the transgenic Tg(Canopy:EGFP) line, which expresses EGFP at the midbrain/hindbrain boundary (MHB), were obtained from stock at the Institute of Cellular and Organismic Biology, Academia Sinica. All the fish lines were reared in a circulating system under a 14/10 h light/dark photoperiod illumination cycle, maintained under standard conditions at 28.5 °C, and staged based on hpf, as previously described [46]. Techniques for the care and breeding of zebrafish have previously been described in detail [47].

### 2.2. Apoptotic Cell Staining

AB strain (wild-type) and *tp*53^M214K^ mutant embryos at the 1- or 2-cell stage were injected with Bad-MO or control-MO (as Bad-M5). The positive control groups were treated with DNase I (0.1 μg/mL) for 4 h. For AO staining, embryos were harvested at 24 hpf and fixed with 4% paraformaldehyde in PBS (pH 7.4) at room temperature for 30 min. The embryos were stained with AO (1 μg/mL) for 3–5 min, washed twice with PBS, and evaluated by fluorescence microscopy (using incident light at 488 nm excitation, with a 515 nm long pass filter for detection) [28]. For the TUNEL assay (In Situ Cell Death Detection Kit, Roche Diagnostics, Indianapolis, IN, USA), the embryos were also fixed in paraformaldehyde at the end of the incubation period (24 hpf) and were then dechorionated and incubated in blocking solution (0.1% H_2_O_2_ in methanol) for 30 min at room temperature to block endogenous peroxidases. Embryos were rinsed with PBST, incubated on ice in a solution of 0.1% Triton X-100 in 0.1% sodium citrate for 30 min to increase permeability, and rinsed again twice with PBST. Afterward, the embryos were incubated with TMR-conjugated nucleotides and terminal deoxynucleotidyl transferase at 37 °C for 1 h. The embryos were analyzed for positive apoptotic cells under a fluorescence microscope equipped with a spot II cool CCD (Diagnostic Instruments, Sterling Heights, MI, USA) [48].

### 2.3. RNA Extraction

To obtain a sufficient quantity of RNA, 30 embryos were pooled as a sample. Samples were homogenized in 0.6 mL of TRIzol Reagent (Invitrogen, Carlsbad, CA, USA). After chloroform extraction, the total RNA samples were purified and treated with DNase I to remove the genomic DNA using an RNeasy Mini Kit (Qiagen, Huntsville, AL, USA). The quantity and quality of total RNA were assessed by A-drop (FastGene) and agarose gel electrophoresis, respectively [48].

### 2.4. Reverse-Transcription Polymerase Chain Reaction (RT-PCR) Analysis

For cDNA synthesis, 5 μg of total RNA was reverse-transcribed in a final volume of 20 μL containing 1 mM dNTPs, 5 μM oligo(dT)_18_, 1 unit of an RNase inhibitor, and 10 units of RevertAid M-MuLV reverse transcriptase (Thermo Scientific, Vilnius, Lithuania) for 30 min, followed by incubation for 60 min at 42 °C. For PCR amplification, 1 μL of cDNA was used as a template in a 20 μL final reaction volume containing 0.25 μM of dNTP, 1.25 units of Ex Taq DNA polymerase (TaKaRa, Shiga, Japan), and 0.2 μM of each primer [48]. The primer sets are given in Table 1.

### 2.5. Quantitative (q)RT-PCR

The mRNA expression levels were measured by qRT-PCR with a Roche Lightcycler Nano (Roche, Penzberg, Germany). The final volume in the well was 20 μL, containing 4 μL of OmicsGreen qPCR 5X master mix (Omics Bio, Taipei, Taiwan), 3.2 ng of cDNA, and 50 nM of primer pairs. The cycling parameters were as follows: 95 °C × 900 s, followed by 45 cycles of 95 °C × 15 s, 60 °C × 20 s, and 72 °C × 20 s. The standard curve of each gene was checked in the linear range, with ß-actin as an internal control [49]. The primer sets are given in Table 2.

### 2.6. Morpholino Oligonucleotides (MOs)

The morpholino-modified antisense oligonucleotides were purchased from Gene Tools (Philomath, OR, USA). The MOs used against *bad* began at (5′- CAGaGATAtTAAAcAtATGTGcCAT-3′). The maximal dosage that caused no obvious toxic effects on embryogenesis was used as follows: Bad-MO at 8 ng/embryo and control-MO (5′-CAGCGATAATAAAGAAATGTGGCAT-3′) at 8 ng/embryo. The MOs were prepared using Danieau solution (58 mM NaCl, 0.7 mM KCl, 0.4 mM MgSO_4_, 0.6 mM Ca(NO3)_2_, and 5.0 mM HEPES; pH 7.6). The MO solution (8 ng/embryo) containing 0.1% phenol red (a visualizing indicator) was injected into zebrafish embryos at the 1-cell stage using a gas-driven microinjector (Medical System Corporation, Greenvale, NY, USA), as previously described. Wild-type embryos without injection were subjected only to Bad protein measurement and morphological observation [48].

### 2.7. Western Blotting

The embryos were injected with *bad* MO and control-MO at the 1-cell stage. A total of 50 embryos were collected at 24 hpf and homogenized in lysis buffer (10 mM Tris-HCl, pH 6.8, 20% glycerol, 10 mM sodium dodecyl sulfate (SDS), and 2% ß-mercaptoethanol). An aliquot of each lysate with 40 μg of protein per sample was separated by electrophoresis on an SDS polyacrylamide gel to resolve the proteins. The gels were immunoblotted with the following antibodies: Anti-bad antibodies (AnaSpec; Cat No:55478), anti-p53 antibodies (GeneTex; Cat No:GTX102965), anti-caspase 8 monoclonal antibodies (BD Bioscience; Cat No:551244), anti-tBid antibodies (Cell Signaling; Cat No:#20025), anti-caspase 3 monoclonal antibodies (Calbiochem; PC66T), anti-Bcl-2 polyclonal antibodies (Cell Signaling; Cat No:#5071), anti-Bcl-xL polyclonal antibodies (AnaSpec; Cat No:554265), and anti-ß-actin monoclonal antibodies (Calbiochem; Cat No:MAB1501), followed by peroxidase-labeled goat anti-mouse secondary antibodies (1:15,000 dilution; Amersham Biosciences, Piscataway, NJ, USA) or peroxidase-labeled goat anti-rabbit secondary antibodies (1:7,500 dilution; Amersham Biosciences, Piscataway, NJ, USA). Chemiluminescence indicative of antibody binding was captured by MultiGel-21 (TOP BIO, Taipei, Taiwan), as previously described [49]. The protein expression level amounts were quantified by a Personal Densitometer (Molecular Dynamic).

### 2.8. Whole-Mount In Situ Hybridization

Fragments of *pax2a* and *fabp7a* were obtained by PCR and inserted into the pGEM-T easy vector (Promega, Madison, WI, USA). The inserted fragments were amplified by PCR using T7 and SP6 primers, and the products were used as templates for in vitro transcription with T7 and SP6 RNA polymerases (Roche) in the presence of digoxigenin (DIG)-UTP (Roche) to synthesize sense and anti-sense probes. Zebrafish embryos were anesthetized on ice and fixed with 4% paraformaldehyde in a phosphate-buffered saline (PBS; 1.4 mM NaCl, 0.2 mM KCl, 0.1 mM Na_2_HPO_4_, and 0.2 mM KH_2_PO_4_; pH 7.4) solution at 4 °C overnight. Afterward, the samples were washed several times for 10 min each with diethylpyrocarbonate (DEPC)-treated PBST (PBS with 0.1% Tween-20). After PBST washing, the samples were incubated with hybridization buffer (HyB, 50% formamide, 5X SSC, and 0.1% Tween 20) at 65 °C for 5 min and with HyB containing 500 μg/mL of yeast tRNA at 65 °C for 2 h before hybridization. After overnight hybridization with 0.5 μg/mL of DIG-labeled antisense or sense RNA probes, the embryos were serially washed with 50% HyB in 2X SSC (at 65 °C for 20 min), 2X SSC (at 65 °C for 10 min), 0.2X SSC (at 65 °C for 30 min, twice), and PBST (at room temperature for 10 min). Afterward, the embryos were immune-reacted with an alkaline phosphatase-coupled anti-DIG antibody (1:8000) and stained with nitro blue tetrazolium (NBT) (Roche) and 5-bromo-4-chloro-3-indolyl phosphate (BCIP) (Roche) for the alkaline phosphatase reaction. The in situ hybridization assay used embryos injected with *bad*-MO and control-MO at 8, 24, and 72 hpf [4,50].

### 2.9. Microarray

Samples from embryos injected with control-MO (Bad-M5) were labeled with Cy3 dye, and samples from embryos injected with Bad-MO were labeled with Cy5 dye. For this purpose, 0.2 μg of total RNA was amplified using q Low Input Quick Amp Labeling Kit (Agilent Technologies, Santa Clara, CA, USA) and labeled with CyDye (Agilent Technologies, USA) during the in vitro transcription process. Next, 0.825 μg of Cy-labeled cRNA was fragmented to an average size of approximately 50–100 nucleotides by incubation with fragmentation buffer at 60 °C for 30 min. Correspondingly fragmented labeled cRNA was then pooled and hybridized to an Agilent Zebrafish Oligo V3 4x44K Microarray (Agilent Technologies, Santa Clara, CA, USA) at 65 °C for 17 h. After washing and drying using a nitrogen gun, the microarrays were scanned with an Agilent microarray scanner (Agilent Technologies, USA) at 535 nm for Cy3 and 625 nm for Cy5. The scanned images were analyzed using Feature Extraction 10.5.1.1 software (Agilent Technologies, USA), employing image analysis and normalization software to quantify the signal and background intensities for each feature and substantially normalizing the data using the rank consistency-filtering LOWESS method. The data were submitted to NCBI for registration (Accession Number: GSE47971). The genes were annotated with Gene Ontology (GO) Tools. The death pathways were analyzed using Pathway Studio 7.1.

### 2.10. Swimming Activity Assay

To determine the swimming activity of zebrafish larvae at 3 dpf, the larvae were tested in slots in an agarose plate (50 mm× 15 mm; length × width) in 5 different groups at 28.5 °C. Individual fish were tested in 5 repeats by stimulation with a brush [44].

### 2.11. Statistical Analysis

Statistical analyses were performed with SPSS 16.0 or GraphPad Prism 8.2.1 software. Bar plots were expressed as the mean standard deviation of at least 3 experimental replicates. Differences between 2 groups were assessed by Student’s *t*-test, and differences between multiple groups were tested by 1-way analysis of variance (ANOVA) followed by Tukey’s post-hoc test. Bars are expressed as mean ± s.d. or mean ± SEM. Statistical significance is shown at * *p* < 0.01; ** *p* < 0.02 and *** *p* < 0.05.

## 3. Results

### 3.1. Bad-Mediated PCD-Regulated Brain Morphogenesis

**Figure 1:**
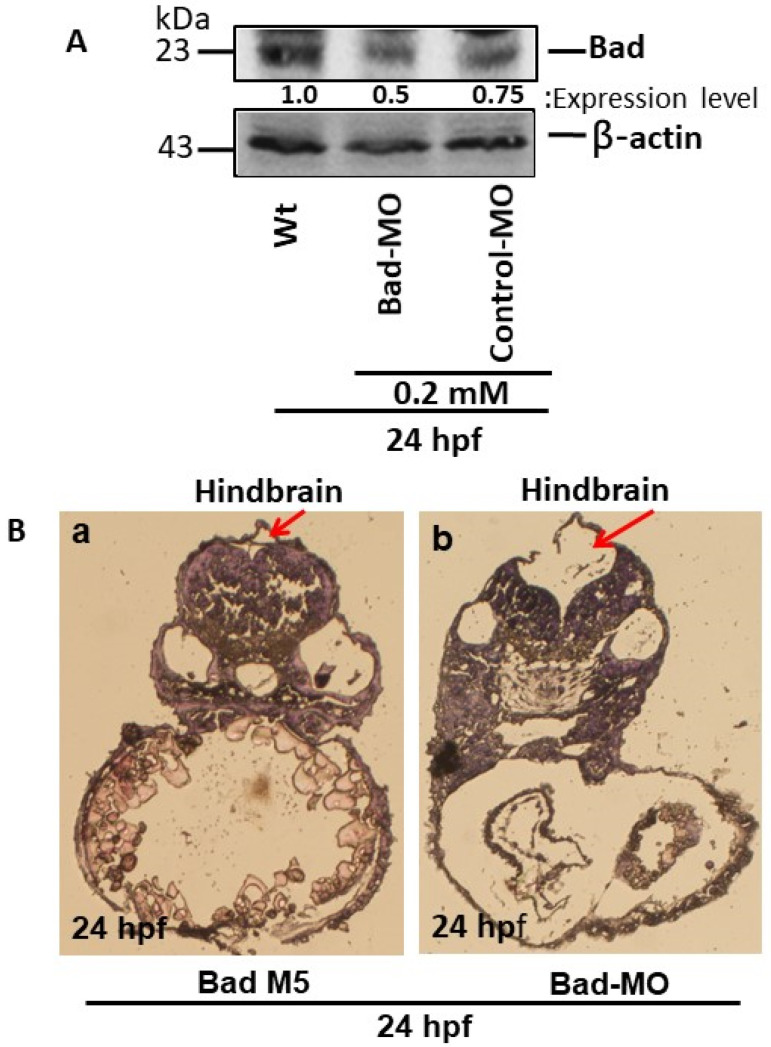
The BH3-only proapoptotic gene Bad can regulate brain development at 24 hpf. (**A**) Bad knockdown (lane 2 for 0.2 mM) shown by Western blot analysis and compared to the uninjected group (lane 1) and the normal control Bad-M5 group (lane 3). The protein expression levels were quantified by a Personal Densitometer (Molecular Dynamics). (**B**) Dissection and observation of brain development in loss-of-Bad-mediated PCD embryos at 24 hpf. HE staining showing hindbrain development in the Bad-M5 group in panel (**Ba**) and the Bad-Mo group in panel (**Bb**) at 24 hpf, as indicated by red arrows.

To define Bad gene function during the early embryonic stage, we adopted a knockdown approach. Using Western blotting, Bad morpholino oligonucleotides (MOs; optima condition as 0.2 mM) effectively knocked down Bad expression at 24 hpf (Figure 1A, lane 2) as compared to the wild-type (Figure 1A, lane 1) and the Bad-M5 control (Figure 1A, lane 3). Then, tissue dissection and hematoxylin and eosin (HE) staining were performed to observe hindbrain morphogenesis. We found that *Bad* knockdown induced midbrain-development defects, as shown in Figure 1Bb (indicated by an arrow), as compared to Bad-M5 (Figure 1Ba). A dramatic malformation was observed in the hindbrain, as shown in the dissection image (Figure 1Bb; indicated by an arrow) [24].

### 3.2. Screening of Bad-Mediated PCD Death Signaling Using a Genome-Wide Approach

**Figure 2:**
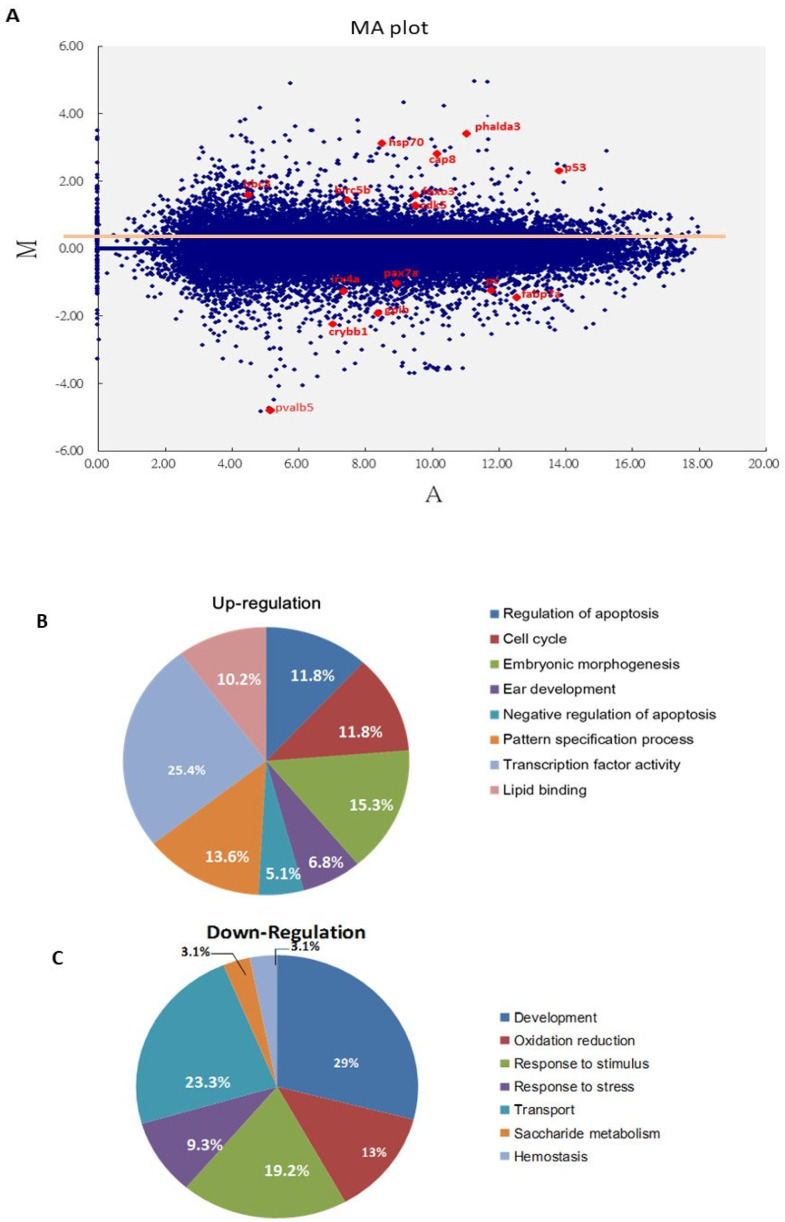
Screening of major cell death pathways via a DNA array approach with Bad knockdown at 24 hpf. (**A**) MA plot showing the distribution of upregulated and downregulated genes in the Bad MO group, with a cut-off of 2 fold changes compared to the Bad M5 group. The key factors p53 and caspase-8 are upregulated. The brain development genes *crybb1*, *pva1b5*, *irx4a*, *pax7a*, and *fabp7a* are marked in red. (**B**,**C**) Changes in biological function genes during loss-of-Bad-mediated PCD are analyzed in separate pie charts for upregulation (**B**) and downregulation (**C**).

To determine which signaling was most strongly involved during Bad knockdown at 24 hpf, the abnormal brain development embryos were corrected for a genome-wide DNA microarray analysis (Figure 2A). Embryos with normal and abnormal brain development were collected, and total RNA was extracted for further analysis. In a genome-wide DNA microarray analysis, we identified up to 2000 genes exhibiting up- or downregulation in the wild-type Bad-MO group, and compared their levels with the wild-type Bad-M5 group, as shown in the MA plot (Figure 2A). The 3 subsets of upregulated genes that strongly induce cell death processes, including the regulation of apoptosis (11.8%), the cell cycle (11.8%), embryonic morphogenesis (15.3%), ear development (6.8%), negative regulation of apoptosis (5.1%), the pattern specification process (13.6%), transcription factor activity (25.4%), and lipid binding (10.2%), are shown in Figure 2B, while the downregulation of 7 gene subsets that can suppress embryonic development and reduce stress responses, including development (29%), oxidation reduction (13%), the response to stimuli (19.2%), the response to stress (9.3%), transport (23.3%), saccharide metabolism (3.1%), and hemostasis (3.1%), are shown in Figure 2C. However, these results require further analysis.

### 3.3. The Role of P53 Function on the Suppression of Apoptotic Cell Death during Bad Knockdown

Based on the DNA array analysis, we further analyzed Bad knockdown-mediated cell death signaling at 24 hpf (Figure 2B). We propose that the p53-mediated stress signal is substantially involved in the Bad-mediated PCD process.

To determine whether p53-mediated death signaling shows major involvement in Bad-mediated PCD during early development, we used *tp*53^M214K^ mutants to examine the loss of its binding capacity [25].

**Figure 3:**
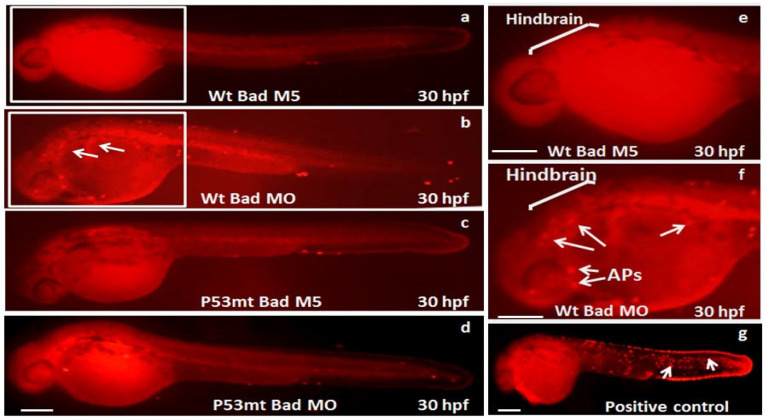
Identification of p53-mediated stress signaling can regulate apoptotic cell death during *Bad* knockdown in p53 mutant (*p53^M214K^*) zebrafish lines. Identification of TUNEL-positive cell death patterns in different fish lines at the 30 hpf stage. In the different fish lines (**a**–**f**), the TUNEL-positive cell deaths (APs), especially in the hindbrain area, are indicated by arrows. The positive control treated with hydrogen peroxide is shown in panel (**g**). Scale bar = 200 μm.

Remarkably, at 30 hpf we found that the TUNEL-positive spots in the p53 mutant line were clearly reduced in the brain area (Figure 3c) compared to the p53 mutant Bad-M5 group (Figure 3d), the wild-type Bad-M5 group (Figure 3a,e), and the wild-type Bad-MO group (Figure 3b,f; indicated by arrows).

### 3.4. Blockage of P53/Caspase-8-Mediated Death Signaling Induction during P53 Loss-of-Function

**Figure 4:**
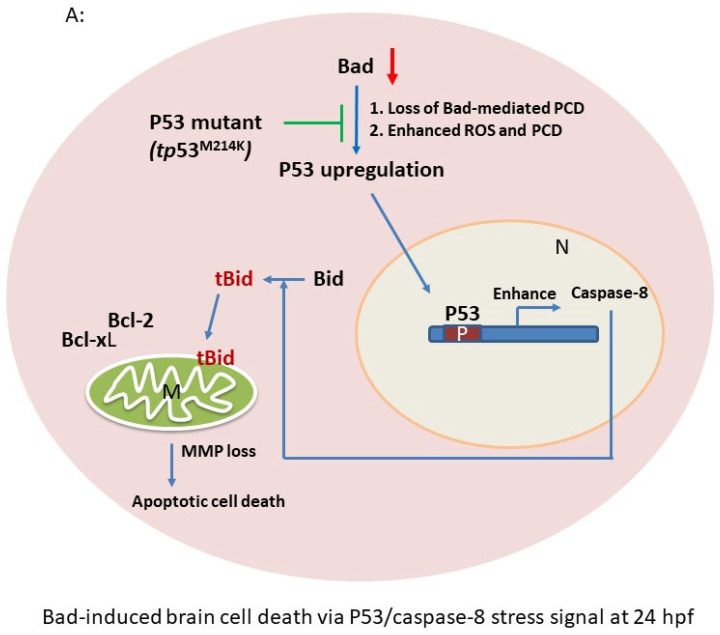

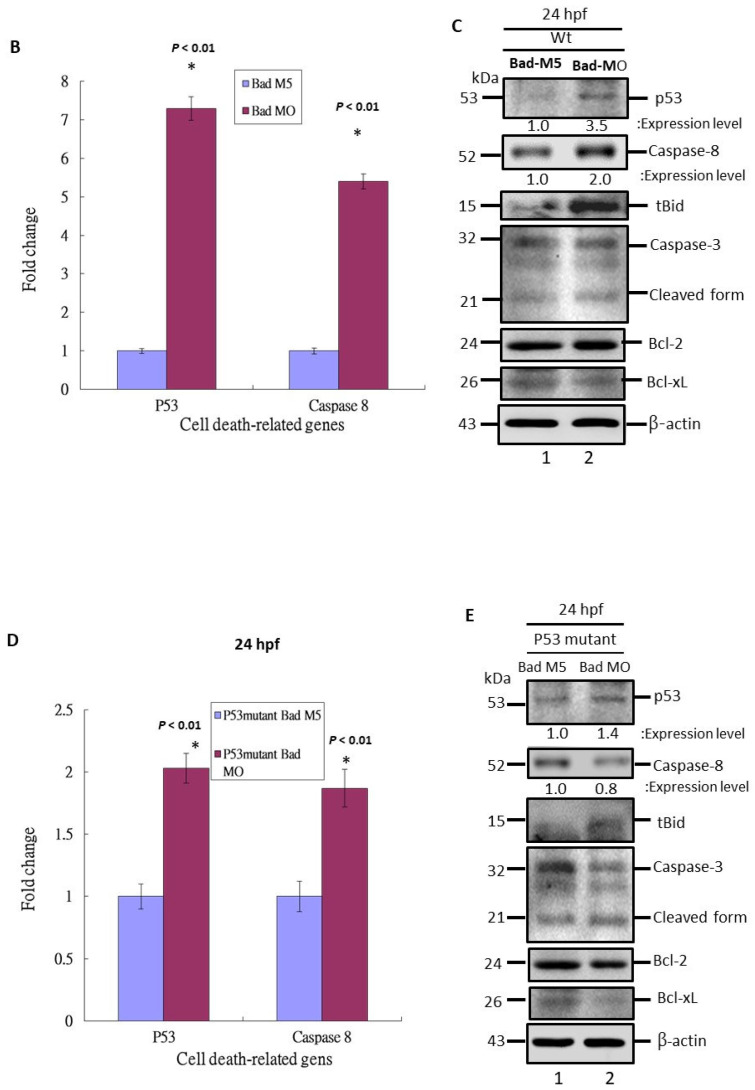
p53/caspase-8 death signaling involvement in *Bad* knockdown embryos at 24 hpf. (**A**) A network signaling death pathway was proposed based on accumulated data. The p53/caspase-8/tBid-mediated death pathway was induced. It also required upstream signaling from loss of Bad, leading to enhanced environmental stress with increased ROS and enhanced apoptotic cell death via the stress/death gene *p53* and its correlated downstream gene expression *caspase-8* or activation of tBid by cleavage from Bid. (**B**) Clear upregulation of *p53* and *caspase-8* demonstrated via qRT-PCR. All data were analyzed using either paired or unpaired Student’s *t*-tests, as appropriate. * *p* < 0.01. (**C**) Identification of apoptotic-related protein expression of p53/caspase-8/tBid as key molecules by Western blotting analysis at 24 hpf. Both p53 and caspase-8 showed stronger expression compared to the control group, correlated with downstream Bid cleavage and anti-apoptotic number Bcl-2 and Bcl-xL expression levels. (**D**) qRT-PCR evaluation of apoptosis-related gene expression with *Bad* knockdown in *p53* mutant lines at 24 hpf. (**E**) Western blot analysis of apoptosis-related protein expression with *Bad* knockdown in *p53* mutant fish lines correlated with downstream protein Bid cleavage and anti-apoptotic number Bcl-2 and Bcl-xL expression levels.

Based on the DNA array analysis, we further analyzed the Bad knockdown-mediated cell death signaling at 24 hpf (Figure 2B and Figure 3). We propose that the p53/caspase-8-mediated apoptotic cell death pathway is substantially involved in the Bad-mediated PCD process (Figure 4A).

First, we evaluated both the *p53* and *caspase-8* genes at the mRNA level by qRT-PCR (Figure 4B), revealing 7- (p53) and 5-fold (caspase-8) enhancement compared to the Bad-M5 group. Then, by Western blot analysis, we found that p53 and caspase-8 were also highly expressed (Figure 4C, lane 2), correlating with the downstream molecule Bid and its further cleavage to the tBid form to interact with the mitochondrial death regulatory factor Bcl-2 or Bcl-xL. This p53/caspase-8-mediated death signaling may activate Bid and Bcl-xL downregulation, but did not cleave caspase-3 from pro-caspase-3 compared to Bad-M5 (Figure 4C, lane 1). The results consistently showed enhanced cell death and reduced embryonic development.

Moreover, upon examining the molecular death mechanism at 24 hpf we found that p53/caspase-8-mediated death signaling was severely reduced in the p53 mutant Bad-MO group (Figure 4D) by up to 5- (*p53* mRNA) and 3-fold (caspase-8 mRNA) compared to the wild-type (Figure 4D) at the mRNA level. The protein levels also showed consistent results (Figure 4E, lane 2) for p53 and caspase-8 and indicated reduced Bid cleavage to tBid compared to the p53 Bad-M5 group (Figure 4E, lane 1).

### 3.5. Loss-of-Function of P53 Enhanced Brain-Developmental Marker Gene Expression

**Figure 5:**
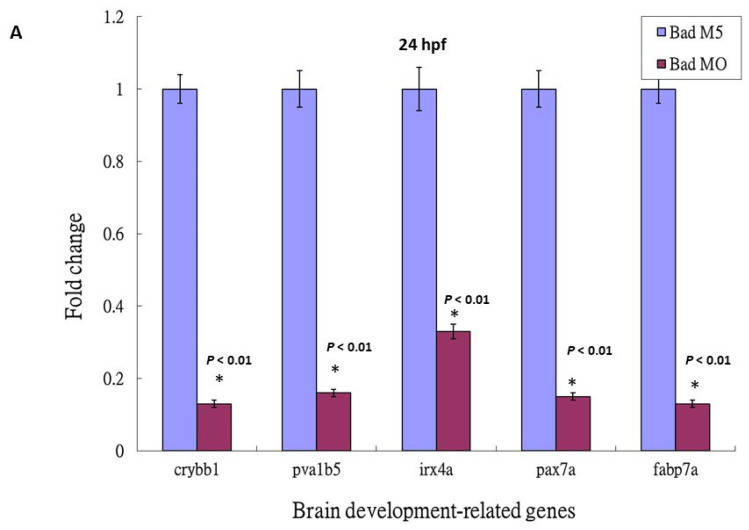

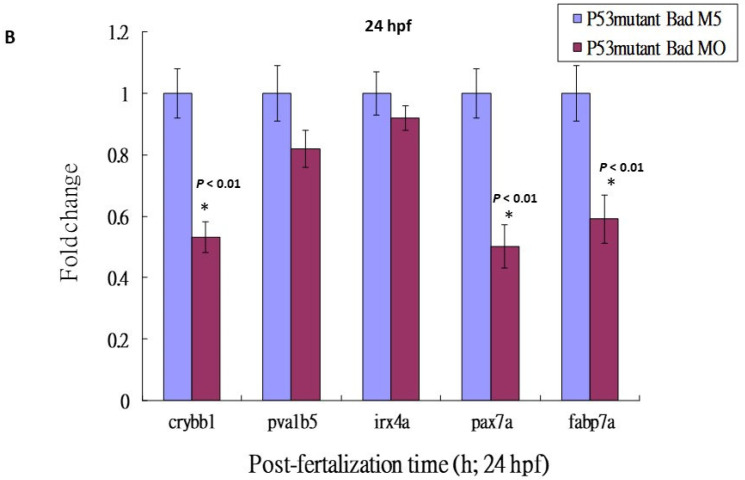
The identification of brain development-related genes was downregulated, but suppressed in the p53 mutant lines with *Bad* knockdown. (**A**) Identification of downstream brain development-related genes such as *crybb1*, *pvalb5*, *irx4a*, *pax7a*, and *fabp7a* in wild-type zebrafish by qRT-PCR at 24 hpf. All data were analyzed using either paired or unpaired Student’s *t*-tests, as appropriate. * *p* < 0.01. (**B**) Enhanced brain-development genes *crybb1*, *pvalb5*, *irx4a*, *pax7a*, and *fabp7a* in p53 mutant lines during Bad knockdown at 24 hpf, as shown by qRT-PCR. All data were analyzed using either paired or unpaired Student’s *t*-tests, as appropriate. * *p* < 0.01.

Based on the DNA array analysis (Figure 2C), we further analyzed the p53-mediated stress signaling during *Bad* knockdown at 24 hpf. This death stress signaling correlated with the effect on brain development: qRT-PCR analysis showed dramatic reductions (Figure 5A) in *crybb1* [51], *pva1b5* [52], *irx4a* [53], *pax7a* [54], and *fabp7a* [55] expression by up to 0.7- to 0.9-fold, confirming the DNA microarray data.

Then, we checked the expression levels of brain-development-related genes such as *crybb1*, *pva1b5, irx4a, pax7a*, and *fabp7a*. The downregulation of these brain development-related genes was clearly suppressed in the p53 mutant Bad-MO group (retaining 0.6- to 0.9-fold expression) and the wild-type Bad-MO group (retaining 0.1- to 0.3-fold expression) (Figure 5B), suggesting that p53-mediated stress signaling is strongly required for complete brain development.

### 3.6. Directly Restoring Hindbrain Development in a P53 Mutant Line

**Figure 6:**
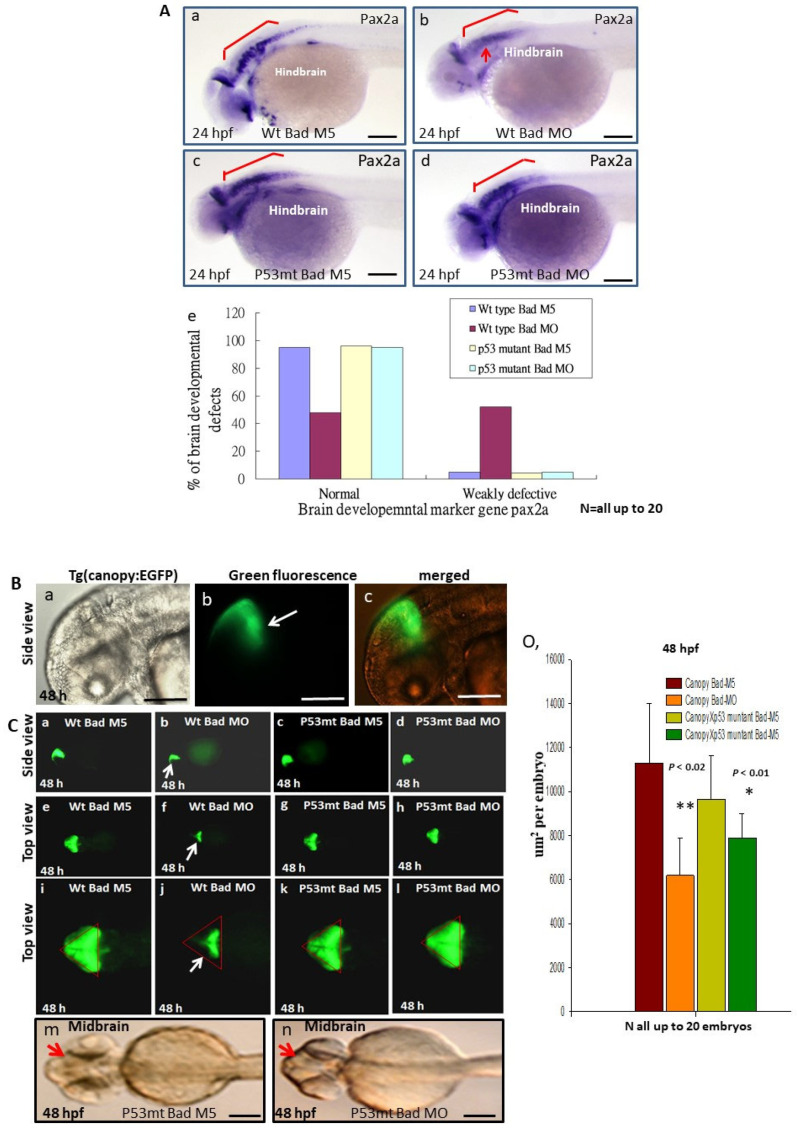

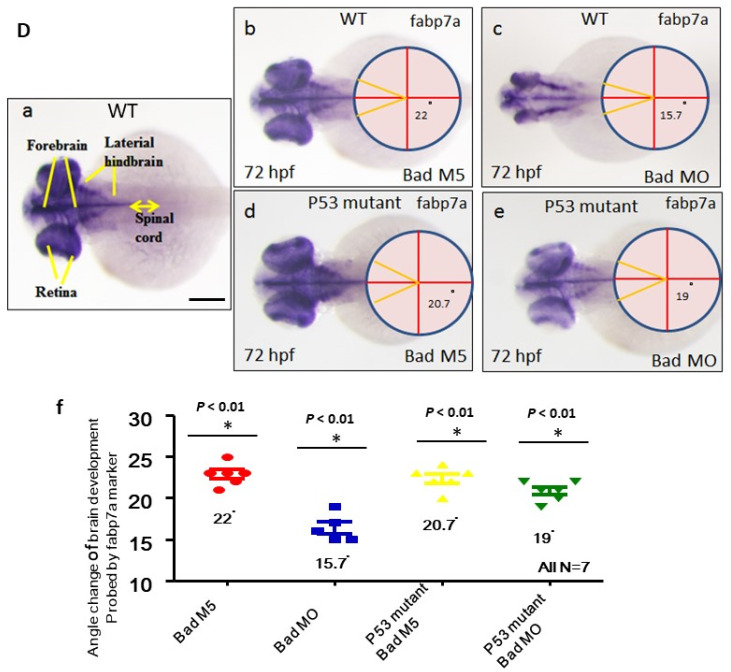
Brain development was directly restored during blockage of the p53-mediated stress signaling transition in p53 mutant lines with *Bad* knockdown. (**A**) Brain developmental marker pax2a expression in an in situ hybridization assay showing normal expression in the p53 mutant line (panels c and d) compared to the wild-type group (panels **Aa**–**Ae**) during Bad knockdown at 24 hpf. Defective marker expression was counted and is shown in panel (**Ae**). Scale bar = 100 μm. (**B**,**C**) The canopy-fused EGFP line was used to monitor brain development directly in the p53 mutant line during loss-of-Bad expression at 48 hpf. The midbrain/hindbrain boundary is indicated by an arrow. (**C**) Blockage of the p53-mediated stress signaling transition can restore brain development (indicated by arrows) compared to the wild-type (panels (**Ca**,**Ce**,**Ci**) for the Bad-M5 group; panels: (**Cb**,**Cf**,**Cj**) for the Bad-MO group) and *p53* mutant lines (panels (**Cc**,**Cg**,**Ck**,**Cm**) for the Bad-M5 group; panels (**Cd**,**Ch**,**Cl**,**Cn**) for the Bad-MO group) at 48 hpf during Bad knockdown. Green fluorescence intensity was recorded and is shown in (**Co**). Scale bar = 200 μm. (**D**) Brain development was probed by used the marker gene *fabp7a* via in situ hybridization in the wild-type and p53 mutant lines. Changes in *fabp7a* marker gene expression at different angles were counted and are shown in (**Df**). Scale bar = 100 μm. All *N* = 7. All data were analyzed using either paired or unpaired Student’s *t*-tests, as appropriate. * *p* < 0.01; ** *p* < 0.02.

To determine whether p53/caspase-8 death signaling is involved in normal development in brain morphogenesis, we used different probes to examine brain development in the p53 mutant line at 24, 48, and 72 hpf. First, at 24 hpf we probed brain development using the *pax2a* (*paired box 2a*) marker gene to detect the mid/hindbrain boundary and hindbrain development (Figure 6A). A 95% normal pattern was observed in the hindbrain area in the wild-type Bad-M5 group (Figure 6Aa,Ae), a 96% normal pattern in the p53 mutant Bad-M5 group (*N* = 23) (Figure 6Ac,Ae), and a 95% normal pattern in the p53 mutant Bad-MO group (*N* = 24) (Figure 6Ad,Ae); however, the wild-type Bad-MO group showed only 52% normal brain patterns (*N* = 28) (Figure 6Ab,Ae; indicated by an arrow).

At 48 hpf, we used a dynamic approach to trace brain development using green fluorescence in the Tg(Canopy:EGFP) zebrafish line. We crossed the canopy-fused EGFP line (Figure 6Ba–Bc) with the p53 mutant line to trace the dynamic development at the midbrain/hindbrain boundary. Interestingly, we observed normal green fluorescent images of the midbrain/hindbrain boundary in the wild-type Bad-M5 group (Figure 6Ca,Ce,Ci), the p53 mutant Bad-M5 group (Figure 6Cc,Cg,Ck), and the p53 mutant Bad-MO group (Figure 6Cd,Ch,Cl); however, the wild-type Bad-MO group (Figure 6Cb,Cf,Cj) showed very severe defects in midbrain/hindbrain boundary conformation and had clearly lost the green fluorescence (Figure 6Cj; indicated by arrow). The total green fluorescence levels from four group embryos were counted and are shown in Figure 6Co, and the results reveal a significant difference. Furthermore, we examined the brain development of the p53 mutant Bad-M5 and p53 mutant Bad-MO groups under phase contrast observation, which appeared to show the same development: Blocked p53-mediated stress signaling during Bad knockdown in a p53 mutant line can restore normal brain development.

At 72 hpf, we used the *fabp7a* (fatty acid binding protein, brain, a) marker gene (Figure 6Ea) to examine the brain development based on the fabp7a signaling pattern at different angles. Bad knockdown in fabp7a expression among the four groups showed marked differences in dynamics. We designed a new method to measure the brain development angle from a lateral view of the hindbrain based on the fabp7a marker pattern (Figure 6D). We found normal development in the p53 mutant Bad-M5 (20.7°; Figure 6Dd,Df), the p53 mutant Bad-MO (19°; Figure 6De,Df), and the wild-type Bad-M5 (22°; Figure 6Db,Df) groups, but wild-type Bad-MO exhibited very severe defects (15.7°; Figure 6Dc,Df) compared to the other three groups, with a difference of approximately 3°, indicating that brain volume was reduced by approximately 20%. All data were analyzed using either paired or unpaired Student’s *t*-tests, as appropriate. * *p* < 0.01.

### 3.7. Restoring Brain Function through the Swimming Ability Test

**Figure 7:**
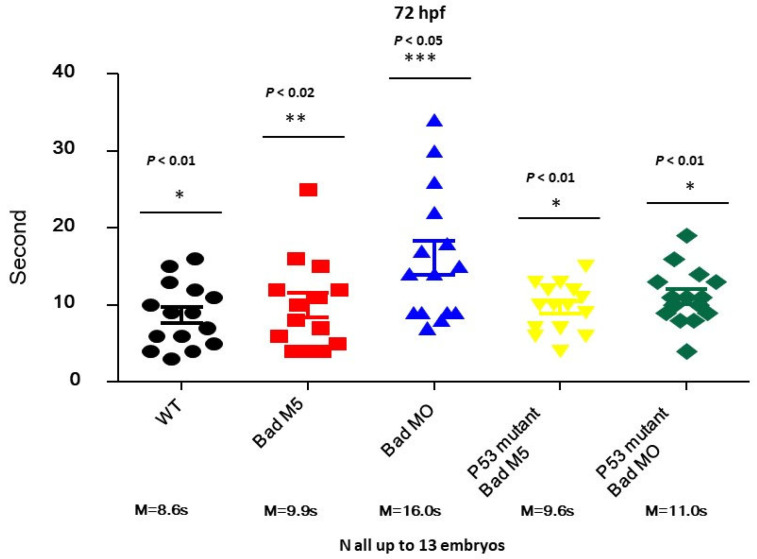
Identification of brain functions through monitoring of swimming behavior at 72 hpf. Swimming speed over a limited length was monitored for different fish lines with and without *Bad* knockdown. The wild-type with Bad-MO group was slower than the other groups by approximately 5 s at 72 hpf. All data were analyzed using either paired or unpaired Student’s *t*-tests, as appropriate. * *p* < 0.01; ** *p* < 0.02; *** *p* < 0.05.

To determine whether p53 can regulate brain function based on swimming ability, we designed a swimming ability test using a slot in agarose gel (1.5 × 50 nm; in Section 2). This test was completed in limited time periods by fishes under different conditions (Figure 7). After testing, we found that the fastest group was the wild-type uninjected group (8.6S), while the second fastest group was the wild-type with Bad-M5 group (9.9S). The third group was the p53mt line with Bad-M5 (9.6S), and the fourth group was the p53mt line with Bad-MO (11.0S). The final group was the wild-type with Bad-MO group (16.0S), which exhibited up to 5 s differences in swimming ability compared to the other groups, further demonstrating that p53/caspase-8 death signaling is required for brain development and brain function based on these statistically significant differences. All data were analyzed using either paired or unpaired Student’s *t*-tests, as appropriate. * *p* < 0.01.

## 4. Discussion

In the zebrafish model system, Bad is interestingly reported as a material factor with expression during the very early embryonic stage (from the one-cell stage to the brain-developmental stage at 24 hpf) [44]. Upon loss of function induced by MOs, the embryos exhibited increased cell death via a TUNEL assay and screening of proteomic profiles. Thus, Bad-mediated cell death is required to regulate either the upregulation of the death genes *p53* and *caspase-8* or the downregulation of brain-development-related genes such as *pavlb5*, *irx4a*, *pax7a*, and *fabp7a* from 24 to 72 hpf. Furthermore, the reduction in p53/caspase-8/tBid death signaling in the p53 mutant line (*p53^M214K^*) enhanced normal brain development and rescued the swimming action during Bad knockdown.

### 4.1. Bad Regulate Caspase-8/tBid Signaling in Brain Development

The recently emerged topic of cell death is taking on new significance. Since the 2000s many studies have focused on the function of Bcl-2 family genes during embryonic development, especially on BH3-only domain members such as Bax, Bak, and Bad in the higher vertebrate mouse system, which possess sequence homology only within the amphipathic α-helical segment that constitutes the critical death domain [13,56].

However, early neural development is not impaired in mice deficient in Bad or mutant mice homozygous for a mutant form of Bad that cannot be phosphorylated in response to survival factors [16,22]. These results suggest that Bad phosphorylation is not essential for regulating survival within early neural precursors.

In our system, we screened the up- and down-regulated genes during *Bad* knockdown at 24 hpf (Figure 2B,C). We postulated the p53/caspase-8 death pathway and tested its potential involvement. Figure 4 show that PCD mediated by the loss-of-Bad function, which correlates with the induction of caspase-8 death signals [57,58,59,60] and further activation of Bid to cleaved tBid [61], can work on mitochondria or regulate downstream caspase-3 activation. In our system, we found that the BH3-only molecule Bad can initiate PCD to reduce micro-environmental stress through ROS induction [44]. Then, Bad-mediated ROS stress can regulate the cell death signaling in downstream events for a more smooth and complete development. In Bad-mediated control, environmental stress is very important and is a newly emerging area.

### 4.2. The Role of the Anti-Tumor-Suppressor P53 on Enhancing Brain Development

In the nervous system, p53 plays a pivotal role in the elimination of new postmitotic neurons that do not differentiate appropriately. Several studies have shown that p53 is involved in the natural cell death of peripheral neurons of the sympathetic superior cervical ganglion soon after birth [62,63,64]. p53 can induce a PCD-signaling cascade after nerve growth factor (NGF) withdrawal and/or p75 neurotrophin receptor activation [62]. p53 overexpression is also sufficient to induce the apoptosis of sympathetic neurons [64,65]. In contrast, p53 inactivation in sympathetic neurons increases cell survival after NGF withdrawal [66,67]. Finally, naturally occurring sympathetic neuronal death is also reduced in p53 mutant mice [62].

Cell death is detected within the neural cells of zebrafish embryos at 12 hpf after the neuroectoderm becomes morphologically distinct [68,69]. Then, cell death is localized within the rostral half of the developing central nervous system (CNS), extending caudally as development proceeds [68,69]. Cell death affects neuronal precursors or newly differentiated neurons before the onset of axon extension, which occurs at 16 hpf [46]. Apoptotic cells are also found in the hindbrain neural folds between E8 and E9 [70]. Then, between E12 and E16, PCD occurs within the ventricular and intermediate zones of the cerebral cortex, which consist of proliferating precursors and newly postmitotic neuroblasts [71,72,73].

Zebrafish are now emerging as a model for complex brain disorders such as depression, autism, psychoses, drug abuse, and cognitive disorders [74]. Depression has been modeled in *gr-s357* zebrafish, which has a mutated glucocorticoid receptor gene [75]. The *gr-s357* zebrafish displays aberrant corticoid biofeedback, including increased levels of glucocorticoids, along with abnormal behaviors related to clinical depression such as reduced locomotion, impaired habituation, and potentiated startle. When administered to *gr-s357* zebrafish, antidepressants such as selective serotonin reuptake inhibitors normalize some of the mutant phenotypes [76]. This zebrafish model illustrates the translational relevance of serotonergic modulation in neurological stress disorders [75]. Using mutagenesis screening, Kim and colleagues recently identified a novel chemokine-like gene family, samdori (sam), involved in mental disorders in zebrafish. Among the five sam family members, *sam2* is specifically expressed in the habenular nuclei of the brain and is associated with intellectual disability and autism spectrum disorder [77]. Using the zebrafish disease model, it was recently found that Armfield X-linked intellectual disability (XLID) syndrome is a form of spliceosomopathy associated with aberrant mRNA processing during development [78,79]. This experiment illustrates the versatility of the zebrafish system for assessing the function of human genes.

**Figure 8:**
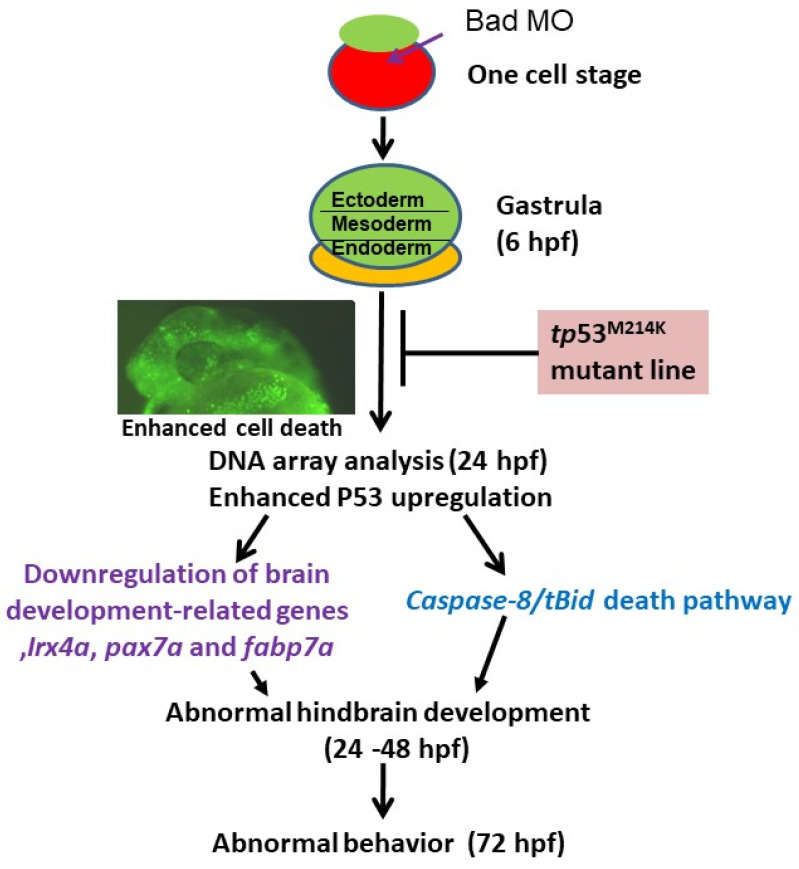
Diagram of Bad’s involvement in committed PCD and brain development from 0.5 to 72 hpf. Early (5.4–12 hpf) PCD is essential for smooth early cell migration and for the formation of the 3 germ layers in later development, which is connected to late (after 12 hpf) PCD for tissue and organ development. Bad-mediated cell death is required for reducing environmental stress, which is correlated with triggering p53-mediated stress signaling on the p53/caspase-8/tBid death pathway and suppression of brain development genes such as *irx4a*, *pax7a*, and *fabp7a*, which are correlated with action behavior. Furthermore, Bad-mediated brain defects should be rescued in the tumor-suppressor p53 mutant (*p53^M214K^*) line. This model system may be a better system for human brain diseases such as autism, psychoses, cognitive disorders, and XLID in that they are correlated with Bad defects.

In our system, we found that Bad knockdown also enhanced apoptotic cell death in the hindbrain at 24 hpf (Figure 1A), which also required p53/caspase-8 upregulation for tBid cleavage (Figure 4C) at 24 hpf. This death signal was apparently blocked during the loss of p53 activity in the p53 mutant line (*p53^M214K^*) (Figure 4E), which restored some important brain and nerve system development genes such as *pavlb5*, *irx4a*, *pax7a*, and *fabp7a* between 24, 48, and 72 hpf. This was very dramatically rescued by directly monitoring in the tg(canopy:EGFP) line, which was localized in the hindbrain area (Figure 6B,C). Furthermore, the molecular biological function in terms of swimming behavior (Figure 7) [2,47] may suggest that Bad is involved in mammalian brain diseases, which are summarized in Figure 8. In the future, genes such as *pavlb5*, *irx4a*, *pax7a*, and *fabp7a* will be further studied during Bad knockdown to analyze their effect on brain function in human diseases such as depression, autism, psychoses, drug abuse, cognitive disorders, and XLID, but this still needs to further examined.

## 5. Conclusions

BH3-only proapoptotic Bad is expressed as a material factor in the early developmental stage, i.e., at 0.5 hpf. If the knockdown of this death factor occurs in the one-cell stage, then at 8 hpf the loss-of-Bad-mediated PCD (PCD begins at 5.4–6 hpf) produces severe environmental stress with heightened ROS production throughout the embryo, which is also correlated with enhanced PCD induction. Interestingly, loss-of-Bad-mediated PCD also affected normal brain development at 24–48 hpf, which correlated with the triggering of p53-mediated stress signaling to regulate both the p53/caspase-8/tBid-mediated cell death pathway and downregulation of brain development-related genes such as *crybb1*, *pvalb5*, *irx4a*, *pax7a*, and *fabp7a*. The p53/caspase-8/tBid-mediated signaling was blocked during p53 function loss, which can directly restore brain development and biological function.

## Figures and Tables

**Table 1:** Primer sequences used for gene cloning.

Primer Sequences for pGEM-T Vector Cloning		
Gene	Forward 5′ → 3′	Reverse 5′ → 3′
*p53*	CTACTAAACTACATGTGCAATAGCAG	CTGAGGCAGGCACCACATCACT
*bad*	ATG GCA CAT ATG TTT AAT ATC TCT GA	CTACTCTGCGGGGCGCGA
*fabp7a*	CTCTCAACATGGTCGATGCATT	CTGGACATTATGCCTTCTCGTA
*pax2a*	CTTCTAACAGGCACATCCCAT	CATTAACCCTCACTAAAGGGAACTATCCGTTCAAAG CCCG

**Table 2:** Primer sequences used for real-time quantitative PCR.

Primer Sequences		
Gene	Forward 5′ → 3′	Reverse 5′ → 3′
*β-actin*	ACT GTA TTG TCT GGT GGT AC	TAC TCC TGC TTG CTA ATC C
*p53*	ACC ACT GGG ACC AAA CGT AG	CAG AGT CGC TTC TTC CTT CG
*Caspase-8*	CCA GAC AAT CTG GAT GAA CTT TAC	TGC AAA CTG CTT TAT CTC ATC T
*pva1b5*	ATGGCACTTGCAGGAATCCTGA	TGTTGGTCTCGGCCTCTGTGAG
*crybb1*	ATGTCTCAGACCGCCAAATCCG	GCCCTGGAAGTTCTCCTGGTCA
*pax7a*	CCAGGAACAGTTCCTCGAATGATG	CCGTGATGGGCCATTTCCAC
*irx4a*	GCGGACAAGGCTACGGGAATT	AGCGTTTTCCTG CGGGTCC
*fabp7a*	TGTGCCACTTGGAAACTGGTTGAC	CCCAGTTTGAAGGAGATCTCGGTG
*Catalase*	TAAAGGAGCAGGAGCGTTTGGCTA	TTCACTGCGAAACCACGAGGATCT
*Mn-sod*	CCGGACTATGTTAAGGCCATCT	ACACTCGGTTGCTCTCTTTTCTCT
*Cu/Zn-sod*	GTCGTCTGGCTTGTGGAGTG	TGTCAGCGGGCTAGTGCTT
*nrf2a*	GAGCGGGAGAAATCACACAGAATG	CAGGAGCTGCATGCACTCATCG
*nrf2b*	GGCAGAGGGAGGAGGAGACCAT	AAACAGCAGGGCAGACAACAAGG

## Data Availability

Not applicable.
